# Effect of attachment configuration and trim line design on the force system of orthodontic aligners: A finite element study on the upper central incisor

**DOI:** 10.1111/ocr.12779

**Published:** 2024-03-09

**Authors:** Tarek M. Elshazly, Christoph Bourauel, Mostafa Aldesoki, Damiano Salvatori, Abdulaziz Alhotan, Ludger Keilig, Ahmed Ghoneima

**Affiliations:** ^1^ Oral Technology, Dental School University Hospital Bonn Bonn Germany; ^2^ Department of Orthodontics and Pediatric Dentistry, Hamdan Bin Mohammed College of Dental Medicine (HBMCDM) Mohammed Bin Rashid University of Medicine and Health Sciences (MBRU) Dubai United Arab Emirates; ^3^ Institut Straumann AG Basel Switzerland; ^4^ Department of Dental Health, College of Applied Medical Sciences King Saud University Riyadh Saudi Arabia; ^5^ Department of Dental Prosthetics, Propaedeutics and Materials Science, Dental School University Hospital Bonn Bonn Germany

**Keywords:** biomechanics, orthodontic force, removable thermoplastic appliance, stress analysis, tooth movement

## Abstract

**Objectives:**

To use the finite element method (FEM) to investigate the effect of various attachment configurations and trimming line designs of orthodontic aligners on their biomechanical performance.

**Method:**

A 3D upper jaw model was imported into 3D design software. The upper right central incisor tooth (Tooth 11) was made mobile, and its periodontal ligament (PDL) and bone structures were designed. Aligners were modelled with three distinct attachment configurations: No attachment, rectangular horizontal, rectangular vertical, and two trimming line designs; scalloped and straight extended, with a homogeneous thickness of 0.6 mm. These models were then imported into an FE software. Simulations were conducted for three different movements, including facial translation, distalization, and extrusion.

**Results:**

Forces were recorded at 1.3–2.6 N during facial translation, 1.4–5.9 N in distalization, and 0.0–2.0 N in extrusion. The straight extended trimming line consistently generated higher forces than the scalloped design. Attachments had no significant impact on force components during facial translation but were more effective in distalization and extrusion. The combination of a straight extended trimming line with horizontal attachments exhibited the least stresses at the apical third during distalization, and the highest stresses during extrusion, suggesting superior retention.

**Conclusions:**

Rectangular attachments offer limited benefits in facial translation, but horizontal rectangular attachments can intensify load in distalization and are crucial for force generation in extrusion. Horizontal attachments are preferred over vertical options. Additionally, the straight extended trim line enhances control of tooth movement and can replace attachments in certain cases.

**Clinical Relevance:**

These findings provide biomechanical evidence and an optimal protocol to guide clinical practice in planning diverse teeth movements. The emphasis is on the influence of attachment utilization and the specific design of aligner trimming lines to enhance control over tooth movement.

**Research Highlights**

- Rectangular attachment provides limited benefits during facial translation.
- Horizontal rectangular attachment increases the load intensity in distalization.
- During extrusion, rectangular attachment is crucial for adequate force generation.
- Horizontally oriented rectangular attachment is favoured over vertical alternatives.
- Straight extended trim line enhances control and can replace attachments in some cases.

## 1 INTRODUCTION

Orthodontic clear aligners are transparent splints custom‐made for patients, facilitating an aesthetically pleasing and popular alternative to traditional braces for teeth correction.^1^ The aligners incrementally move teeth based on a predetermined design, generating adequate forces for teeth alignment.^2^, ^3^, ^4^ Several studies have been conducted to evaluate the biomechanical behaviour of orthodontic aligners and to identify ways to improve their effectiveness and performance through the use of new materials, designs, and technologies.^5^, ^6^, ^7^, ^8^, ^9^, ^10^ Despite extensive research on aligner biomechanics, discrepancies between planned and actual results persist.^11^, ^12^ Moreover, a definitive geometry for optimizing aligner modifications to accommodate various tooth movements has not been endorsed. Hence, further studies are needed to enhance the evidence and predictability of bodily movement (Translation) and torque control with aligners.^13^

The accuracy of orthodontic aligner treatment was reported to be affected by many factors such as aligner material,^14^ activation and staging,^15^, ^16^, ^17^ thickness,^18^ and edge extension and trimming design.^19^, ^20^, ^21^, ^22^ However, in light of the mechanisms governing orthodontic aligner functionality, not all forms of tooth movement can be feasibly achieved solely through the application of aligners.^12^, ^13^ Consequently, the incorporation of supplementary auxiliaries and attachments appears imperative in order to attain desired treatment outcomes.^4^, ^8^, ^13^, ^17^, ^23^ The literature has documented various shapes for attachments, such as rectangular, rectangular bevelled, and ellipsoid.^24^ Yet, the rectangular attachments are the most prevalent and favourable choice in practice.^25^ Nonetheless, the position of the attachment was reported to be more critical than the shape of the attachment to avoid undesired movements.^17^

Orthodontic treatment relies on the pressure‐tension theory that involves the interaction of teeth, the periodontal ligament (PDL), and the alveolar bone. However, mimicking this complex process in experimental studies is challenging due to the absence of PDL and bone elements.^26^ Alternatively, the finite element method (FEM) is a non‐destructive, repeatable, and efficient method broadly used to investigate the biomechanics of orthodontic appliances and clear aligners.^2^, ^11^, ^27^, ^28^ It is an accurate numerical engineering tool used to solve complex analytical equations. In FEM, the analysed domain is divided into a finite number of smaller subdomains (elements), and the complex equations are solved over each element. Using the solutions from the individual elements, the solution of the whole domain is then pieced together to get the overall analysis.^29^

The current FE model is employed to investigate different orthodontic treatment strategies by clear aligners, which may be challenging to visualize through experimental means. The model's design, sensitivity analysis, parameters selection, and validation have been documented in earlier reports.^22^, ^27^ The main upgrade to the present model involves incorporating PDL and bone for more realization, as well as horizontal and vertical rectangular attachments in some designs. The focus is on assessing the potential impact of rectangular attachment use and orientation on various movements of the upper central incisor tooth, namely facial translation, extrusion, and distalization. This integration is done alongside two distinct trimming line designs, scalloped and straight extended, to investigate whether modifying the trimming line design could substitute for attachments in specific treatment scenarios.

## 2 MATERIALS AND METHODS

**FIGURE 1:**
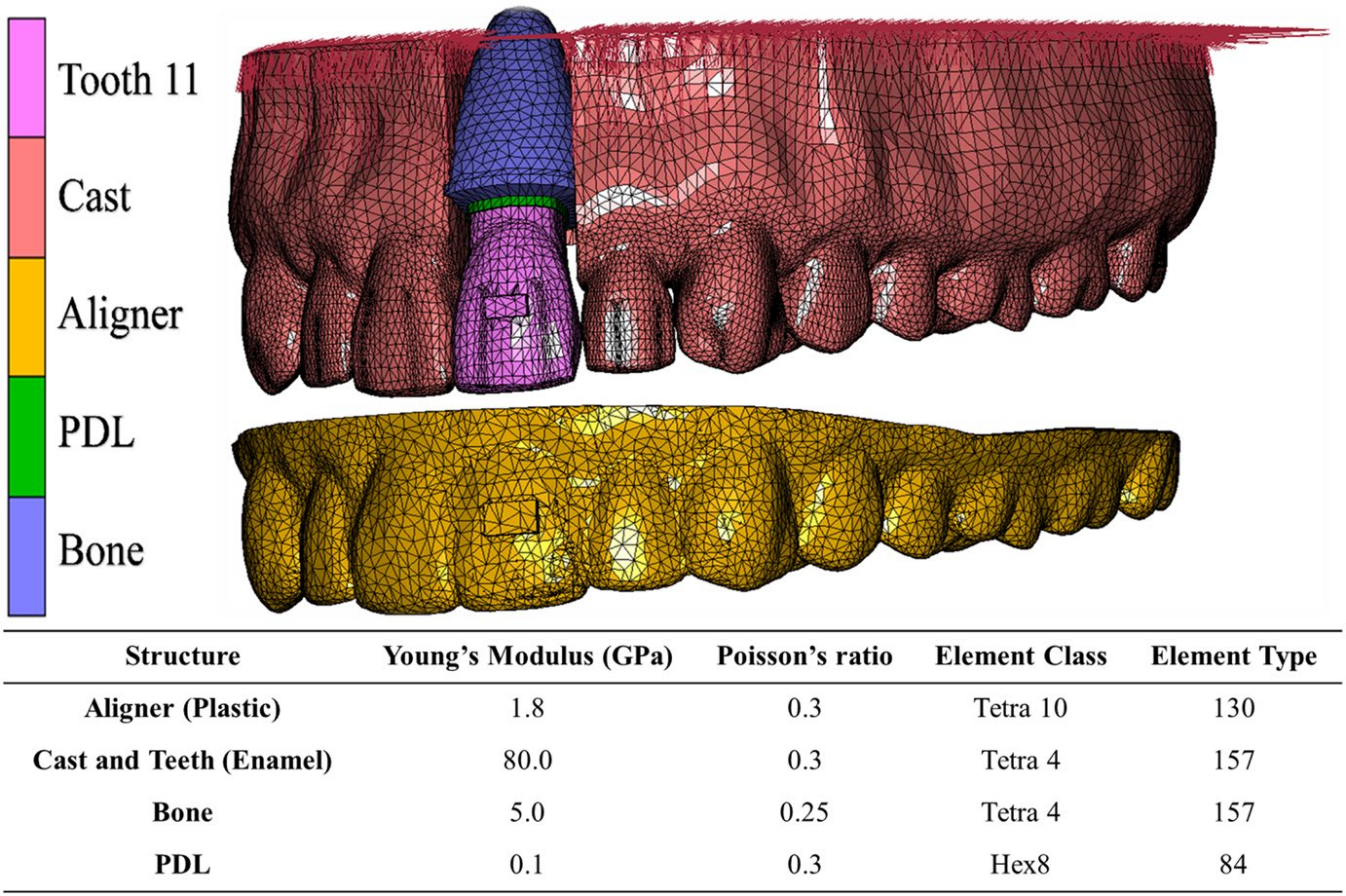
A digital 3D model for finite element analysis. The table shows the material parameters and elements given to each object.

**FIGURE 2:**
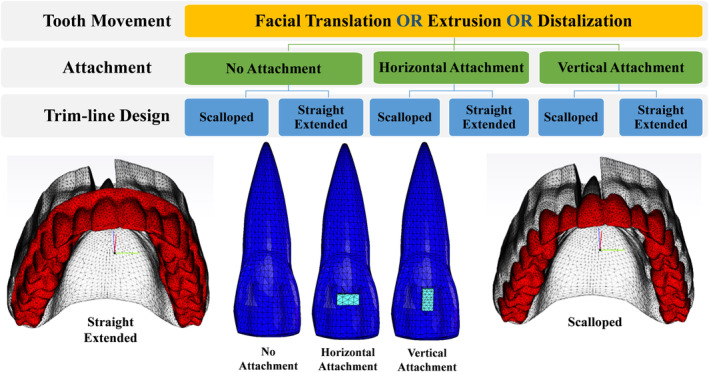
A schematic illustration of the current study design, and the digital 3D models of orthodontic aligners with different trimming line designs and different attachment models.

Software for image processing and editing (3‐matic 16.0; Materialize, Leuven, Belgium) was used to create a digital model of an upper arch from a 3D data set of a maxilla (Digimation Corp., St Rose, Louisiana, USA). The upper right central incisor (Tooth 11) was made individually moveable, and the periodontal ligament (PDL) and bone were designed around it. Both the PDL and bone had uniform thicknesses of 0.2 and 2 mm, respectively (Figure 1). This model has been well described, validated, and used in recent studies.^22^, ^27^ However, certain adjustments were made to suit the specific objectives of the current study, in which there are a total of 18 distinct study groups categorized according to variations in aligner trimming line design, attachment utilization, attachment orientation, and direction of movement of Tooth 11, as visually represented in Figure 2.

In 3‐matic, a rectangular attachment measuring 4 × 2 × 2 mm was created at the centre of the facial surface of the crown of Tooth 11 in two orientations: horizontal and vertical, as shown in Figure 2. Moreover, aligners were modelled with a homogenous thickness of 0.6 mm, in two different trimming line designs: scalloped (at the level of the gingival line) and straight extended (with a straight 2 mm extension beyond the gingival line) (Figure 2).

The various models were imported into an FE software (Marc/Mentat 2015; MSC Software, Los Angeles, California). In Marc/Mentat, the surface elements of the model were remeshed and converted into solid elements (Figure 1). The specifics of the element and node types and numbers for each part of the model are listed in Supplement S1. The linear elastic constitutive material model and the material parameters given to different model parts were defined (Figure 1).^27^, ^30^, ^31^ A glue contact interaction was used to join the bone‐PDL and tooth‐PDL interfaces, while a touching mode was used between aligner and teeth without defining friction and with a (−0.04) interference closure. Any contact between the cast and the Tooth 11 was ignored.

A local coordinate system was defined for Tooth 11, separate from the coordinate system of the cast,^27^ in which the X‐axis is pointed to the facio‐lingual direction, the Y‐axis is pointed to the mesio‐distal direction, and the Z‐axis is pointed to the intrusion–extrusion direction, with the positive directions pointing towards the facial, mesial, and intrusion of Tooth 11. The lower surface nodes of the cast were immobilized in all three translation directions. Furthermore, the nodes at the distal ends of the aligner were similarly fixed to ensure numerical stability during the simulation of the model. The tooth movement was simulated using selected nodes at the apical and proximal outer surface of the bone. Displacement in the three directions of movement was a linearly increasing movement up to a maximum displacement of 0.2 mm in each direction, while it was constrained in the other two directions. The calculations were run on a high‐performance computing workstation (Dell, Round Rock, Texas, USA).

## 3 RESULTS

**FIGURE 3:**
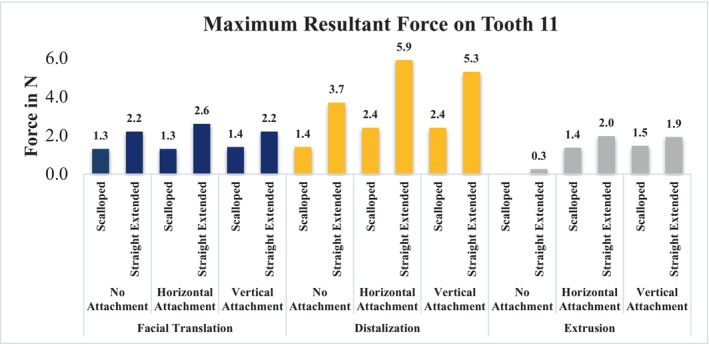
Initial maximum resultant force (in N) generated on an upper central incisor in different directions of 0.2 mm movement.

Figure 3 displays the maximum resultant forces applied by aligners, considering various trimming line designs and different attachment configurations, when a 0.2 mm displacement of Tooth 11 occurs in different directions. The forces ranged from 1.3 to 2.6 N during facial translation, from 1.4 to 5.9 N in distalization, and from 0.0 to 2.0 N in extrusion. Notably, forces were consistently higher with the straight extended trimming line design compared to the scalloped design. Attachments did not significantly affect force generation during facial translation, while they enhance force during distalization. On the contrary, during extrusion, use of attachments is essential for adequate force generation, with no notable difference between the horizontal and vertical configurations in this regard.

**FIGURE 4:**
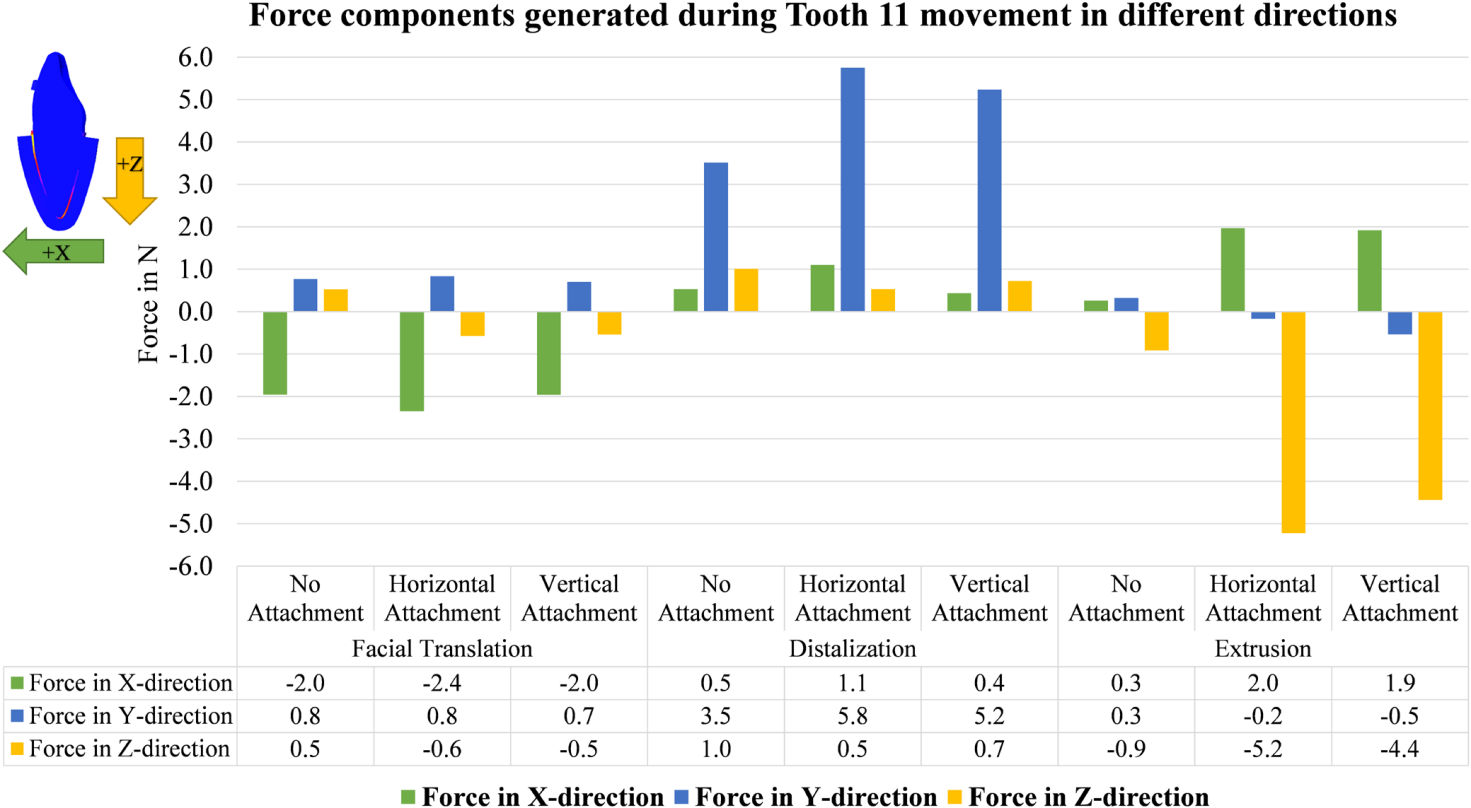
Force components (in N) generated on an upper central incisor in different directions of movement (Facial translation, Distalization, and Extrusion; 0.2 mm), the green and yellow arrows indicate the direction of bone movement during the simulation.

Figure 4 provides a thorough representation of the force components during various directional movements. During facial translation (X‐axis), undesirable forces in Y‐ and Z‐axes occur. Similarly, in distalization (Y‐axis), undesirable forces in X‐ and Z‐axes occur, and in extrusion (Z‐axis), undesirable forces in X‐ and Y‐axes occur. During facial translation, the use of attachments did not yield a significant noticeable variance. During the distalization process, the horizontal attachment design demonstrated the highest force generation with slight unwanted forces along the X (lingualization) and Z (intrusion) axes. However, its performance closely resembled that of the vertical attachment design. During extrusion, attachment‐free design resulted in minimal force, while horizontal attachments showed favourable force profiles, closely resembling vertical attachment performance with no significant differences.

**FIGURE 5:**
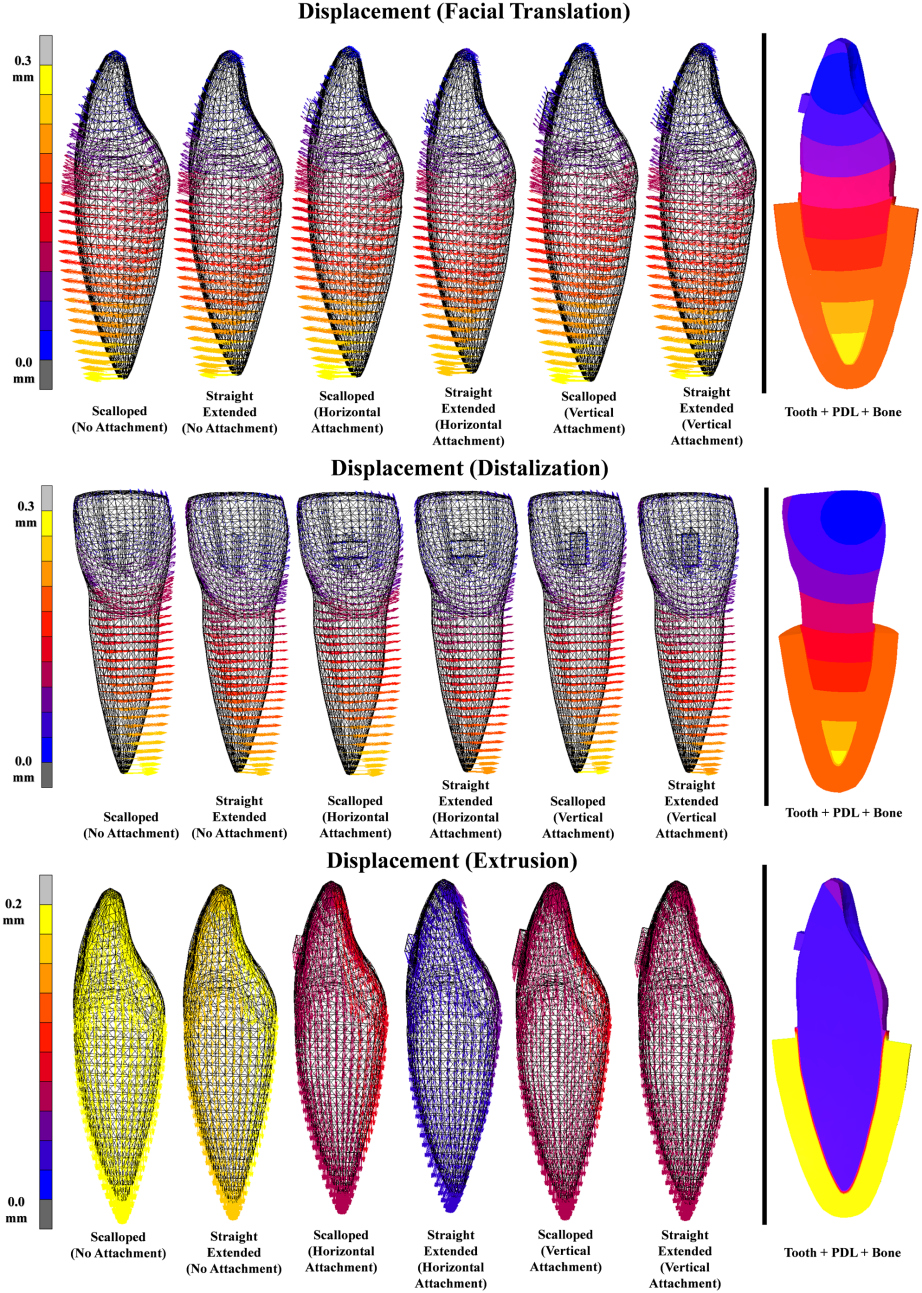
Initial displacement vector of an upper central incisor tooth. The FE simulation is commenced by introducing a 0.2 mm displacement of the bone in the intended direction, eliciting corresponding responses from the tooth and the PDL.

In Figure 5, greater aligner retention correlates with reduced tooth mobility, improving control over displacement. During facial translation, no discernible differences were observed among groups; uncontrolled tipping behaviour was noted. Despite targeting a 0.2 mm displacement, the root apex shifted by 0.3 mm, indicating partial compression of the PDL by 0.1 mm. Similarly, during distalization, no significant differences were observed; slight rotation and 0.1 mm partial compression in the PDL at the root apex were noted. For extrusion, the combination of a straight extended trimming design with a horizontal attachment showed the highest retention and constrained tooth movement, while the attachment‐free design exhibited minimal retention, limiting control over extrusion, as evidenced by minimal force generation data in Figures 3 and 4.

**FIGURE 6:**
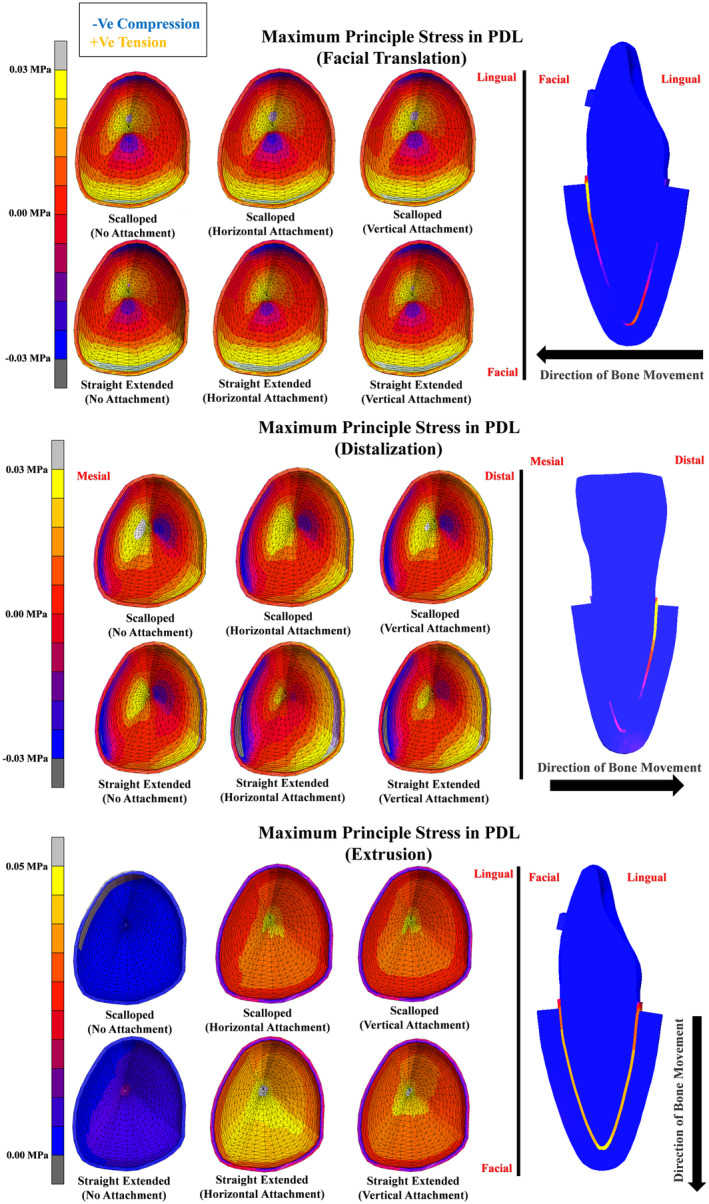
The initial maximum principle stress in PDL of an upper central incisor on a 0.2 mm displacement.

In Figure 6, the maximum principal stress in PDL of Tooth 11 is demonstrated, with positive indicating tensile stresses and negative indicating compressive stresses. During facial translation, no noticeable differences were observed among groups. Tensile stresses peaked at 0.04 MPa on the facial‐cervical and lingual‐apical areas, while compressive stresses reached −0.03 MPa on the facial‐apical and lingual‐cervical regions, reflecting the tipping phenomenon in Figure 5. During distalization, stress distribution aligned with tip movement, showing rotation between the mesial and distal root halves. The straight extended trimming line design with horizontal attachment minimized stresses at the apical third. Similarly, during extrusion, stress distribution mirrored Figure 5, with attachment‐free designs experiencing minimal stress and the combination of the straight extended trimming line design and horizontal attachment showing the highest stresses, indicating superior retention.

## 4 DISCUSSION

Aesthetics plays a pivotal role in patients choosing aligner treatments, and excessive or large composite attachments on the buccal surface of teeth can compromise treatment invisibility and, consequently, patient acceptance.^32^ Hence, it is crucial to refine the design of aligner treatments, minimizing attachments while preserving the aligner efficiency.

In a clinical context, aligners are typically programmed with a discrepancy relative to the planned tooth position. This programming is intended to establish a force system that will guide the tooth towards the desired location. However, in the present FE study, the opposite was simulated, where the aligner design initially matched the aligned dentition, and the mismatch was introduced by displacing Tooth 11 from its aligned position to a 0.2 mm malposition in different directions (facial translation, distalization, and extrusion). This approach avoided the complicated simulation of placing the aligner onto the dental arch with a malpositioned tooth, while it does provide a clear and controlled means to calculate the initial force system exerted by an aligner on a misaligned tooth.^27^ Furthermore, although the PDL has non‐linear elastic material behaviour, previous studies^2^, ^33^ have suggested that the rigidity of teeth and bone is much higher than that of PDL, and hence assuming linearity in FE analysis will not significantly impact the final outcomes. Moreover, Cattaneo et al.^30^ have also argued that the non‐linear response of the PDL may not be necessary when investigating the initial phase of orthodontic treatment, as is simulated in the present study.

The force values documented in the present study notably exceed the recommended orthodontic force values (0.5–1.5 N).^34^ However, aligners in use tend to diminish force levels substantially within a few hours, reaching residual values of approximately 10%–20% after few days.^35^ Consequently, an initial force up to 6 N is acceptable.

Consistent with the findings in consecutive experimental and numerical investigations conducted by Elshazly et al.,^21^, ^22^ the use of an aligner with a straight extended trimming line design led to improved bodily tooth movement control, enhanced stress distribution, and the application of greater force closer to the gingival region, which is closer to the centre of resistance, in favour of bodily translation. Similar findings were reported by Brown et al.^20^ and Gao et al.^19^

In one line with Simon et al.^36^ and Costa et al.,^37^ the clear aligners generally do not promote purely translational tooth movement but tend to generate undesired forces on the teeth. Furthermore, the inclusion of a rectangular attachment did not totally mitigate these undesirable forces, consistent also with Ayidaga et al.^11^ and Ho et al.^38^ On the contrary, the influence of attachment use, especially when employing an extended straight trimming line design, exhibits varying effects upon meticulous examination of different tooth movements.

During facial translation of Tooth 11, the dominant movement is uncontrolled tipping, in agreement with the findings of Elkholy et al.,^13^ and the current FE model showed that incorporating attachments did not significantly alter force levels. This supports the idea that aligners' clinical effectiveness in achieving specific tooth movements relies more on precise planning of movement increments and careful selection of aligner material than solely on attachment use.^13^, ^38^, ^39^

In distalization, employing an attachment‐free configuration with a straight extended trimming line design helps maintaining acceptable force levels. However, the present force analysis suggests that incorporating rectangular attachments, particularly when oriented horizontally, improves the effectiveness of tooth distalization by augmenting applied force levels in the intended direction, as reported in a recent review^13^ and validated by a previous clinical study.^40^ Furthermore, the combined application of a rectangular attachment and a straight extended trimming design induces a greater distal displacement of the root apex, as evidenced by the reduction in stress levels at the apical portion of the root. This effect has the potential to contribute to the reduction of root resorption. Similarly, Gomez et al.^28^ proposed that the presence of attachments is more conducive to bodily tooth movement, since they generate a counteracting force that mitigates the inclination for tipping when an aligner segment is distally displaced in the absence of attachments. Also, Yokoi et al.^31^ demonstrated that the attachments were effective for achieving bodily movements of central incisors during diastema closure.

Regarding extrusion, the accuracy of tooth movement is the lowest in the absence of attachment use, mainly due to a lack of secure grip by the aligner on the target tooth during vertical pulling. In an attempt to address these challenges, the incorporation of attachments was recommended.^10^ The current extrusion simulations showed that the incorporation of a horizontal rectangular attachment showed superior performance in both movement patterns and force levels, especially when used with the straight extended trimming line design. These outcomes align with the findings of FE studies conducted by Savignano et al.^25^ and Laohachaiaroon et al.,^24^ as well as the experimental study by Costa et al.^37^ for Tooth 11. Additionally, the current force values recorded during extrusion with attachment configurations (approximately 2.0 N) are consistent with their values. The analysis of force revealed the presence of an undesired force in the X‐direction, resulting in a lingual crown‐tipping moment. This occurs because the force applied during extrusion is directed towards the cervical segment of the rectangular attachment, located on the facial side of the centre of resistance of Tooth 11.

The present study has the limitation of only simulating the initial state of Tooth 11 movement in a unidirectional manner, without accounting for bone remodelling during aligner treatment. Additionally, the model calculated only 3D forces due to limitations in accurately computing moments with the current tetrahedral elements. Moreover, controlling vertical alignment in clear aligner therapy, especially accounting for the potential “bowing effect” during certain teeth movements, remains challenging,^8^ but the current study is limited in exploring this effect due to the absence of PDL and bone structure around teeth, except for Tooth 11. Future research should involve various teeth with intact PDL and bone structures. Also, incorporating numerical simulation of bone resorption is essential. While FEM provides reliable stress analysis, discrepancies can exist between numerical simulations and real clinical results. Therefore, meticulous, high‐quality clinical investigations are crucial to validate force systems derived from FEM.

## 5 CONCLUSIONS

According to the current FE model outcomes, the following conclusions can be drawn concerning movement of Tooth 11 by aligners:

1. During facial translation, employing a straight extended trimming design leads to enhanced control and can serve as a viable alternative to attachments, with the addition of rectangular attachments offering minimal additional benefits.
2. During distalization, the horizontal rectangular attachment increases the force intensity in the distal direction.
3. During extrusion, the utilization of rectangular attachments appears to be essential in achieving the necessary force levels.
4. Upon closer examination of the force analysis, there is a minimal distinction between the horizontal and vertical rectangular attachments, with a preference for the horizontal configuration.
5. For distalization and extrusion, the combination of a straight extended trimming line and a horizontal rectangular attachment demonstrates superior performance, particularly in terms of principal stress distribution within the periodontal ligament, displacement patterns, and force generation levels.

## AUTHOR CONTRIBUTIONS

Conceptualization: TE, CB, and DS. Data curation and Analysis, Investigation, and Methodology: TE, CB, DS, and LK. Resources: CB, DS, AA, and AG. Software: TE, CB, MA, and LK. Supervision, Validation and Visualization: CB, DS, LK, and AG. Writing—original draft: TE. Writing—review & editing: TE, CB, MA, DS, AA, LK, and AG. All authors have read and agreed to the published version of the manuscript.

## FUNDING INFORMATION

Part of this work was supported by Straumann AG, Basel, Switzerland.

## CONFLICT OF INTEREST STATEMENT

The authors declare that they have no competing interests.

## Supporting information


Data S1.

## Data Availability

The datasets used and/or analysed during the current study are available from the corresponding author on reasonable request.
