# Supplementary material for: Effect of attachment configuration and trim line design on the force system of orthodontic aligners: A finite element study on the upper central incisor
Source: Orthod Craniofac Res. 2024 Mar 9;27(Suppl 2):131–40. doi: 10.1111/ocr.12779 (PMC11654353; doi:10.1111/ocr.12779)
Supplement: Supplementary file 1 — Data S1. [file OCR-27-131-s001.docx]

Supplement 1: Class and type of elements, as well as the number of elements and nodes of different bodies of the finite element model used in the current study.

| Object | Element Class, Element Type | Number of Elements | Number of Nodes |
| --- | --- | --- | --- |
| Tooth 11 with no attachment | Tetra 4, type 157 | 10407 | 1730 |
| Tooth 11 with horizontal attachment | Tetra 4, type 157 | 11283 | 2841 |
| Tooth 11 with vertical attachment | Tetra 4, type 157 | 11706 | 2928 |
| Cast | Tetra 4, type 157 | 174185 | 23746 |
| Periodontal Ligaments (PDL) | Hex 8, type 84 | 5587 | 1719 |
| Bone | Tetra 4, type 157 | 9635 | 2265 |
| Scalloped aligner (no attachment) | Tetra 10, type 130 | 81267 | 84970 |
| Scalloped aligner (horizontal attachment) | Tetra 10, type 130 | 83005 | 154268 |
| Scalloped aligner (vertical attachment) | Tetra 10, type 130 | 83316 | 154752 |
| Straight extended aligner (no attachment) | Tetra 10, type 130 | 52952 | 61162 |
| Straight extended aligner (horizontal attachment) | Tetra 10, type 130 | 53468 | 102386 |
| Straight extended aligner (vertical attachment) | Tetra 10, type 130 | 54544 | 104162 |
